# MoDorado: enhanced detection of tRNA modifications in nanopore sequencing by off-label use of modification callers

**DOI:** 10.1093/nar/gkaf795

**Published:** 2025-08-19

**Authors:** Franziskus N M Rübsam, Wang Liu-Wei, Yu Sun, Bhargesh Indravadan Patel, Wiep van der Toorn, Michael Piechotta, Christoph Dieterich, Max von Kleist, Ann E Ehrenhofer-Murray

**Affiliations:** Institut für Biologie, Lebenswissenschaftliche Fakultät, Humboldt-Universität zu Berlin, 10115 Berlin, Germany; Systems Medicine of Infectious Disease (P5), Robert Koch Institute, 13353 Berlin, Germany; Department of Mathematics and Computer Science, Freie Universität Berlin, 14195 Berlin, Germany; International Max-Planck Research School ‘Biology and Computation’, Max-Planck Institute for Molecular Genetics, 14195 Berlin, Germany; Institut für Biologie, Lebenswissenschaftliche Fakultät, Humboldt-Universität zu Berlin, 10115 Berlin, Germany; Institut für Biologie, Lebenswissenschaftliche Fakultät, Humboldt-Universität zu Berlin, 10115 Berlin, Germany; Systems Medicine of Infectious Disease (P5), Robert Koch Institute, 13353 Berlin, Germany; Department of Mathematics and Computer Science, Freie Universität Berlin, 14195 Berlin, Germany; Klaus Tschira Institute for Integrative Computational Cardiology, University Hospital Heidelberg, 69120 Heidelberg, Germany; Department of Internal Medicine III (Cardiology, Angiology, and Pneumology), University Hospital, 69120 Heidelberg, Germany; Klaus Tschira Institute for Integrative Computational Cardiology, University Hospital Heidelberg, 69120 Heidelberg, Germany; Department of Internal Medicine III (Cardiology, Angiology, and Pneumology), University Hospital, 69120 Heidelberg, Germany; German Centre for Cardiovascular Research (DZHK)-Partner Site Heidelberg/Mannheim, 69120 Heidelberg, Germany; Systems Medicine of Infectious Disease (P5), Robert Koch Institute, 13353 Berlin, Germany; Department of Mathematics and Computer Science, Freie Universität Berlin, 14195 Berlin, Germany; Institut für Biologie, Lebenswissenschaftliche Fakultät, Humboldt-Universität zu Berlin, 10115 Berlin, Germany

## Abstract

Rapid and accurate identification of transfer RNA (tRNA) modifications is crucial for understanding their role in protein translation and disease. However, their detection on tRNAs is challenging due to high modification density. With the release of the nanopore direct RNA sequencing kit SQK-RNA004, *de novo* modification calling models became available for pseudouridine (Ψ), m^6^A, inosine, and m^5^C, as part of the Dorado basecaller. By applying the Ψ model to tRNAs, we mapped both known and previously uncharacterized Ψ sites in *Schizosaccharomyces pombe*, and identified the corresponding pseudouridine synthases. This led to the discovery of two novel modification sites, Pus7-dependent Ψ8 and Pus1-dependent Ψ22. Furthermore, we have developed MoDorado, an algorithm to detect modifications beyond those used in model training (“off-label use”). It does so by assessing differences in modification predictions between modified and nonmodified samples using pre-trained, modification-specific models. By repurposing the Ψ/m^6^A/inosine/m^5^C models, MoDorado detected seven additional modifications (ncm^5^U, mcm^5^U, mcm^5^s^2^U, m^7^G, queuosine, m^1^A, and i^6^A), thus generating an expanded map of these tRNA modifications in *S. pombe*. This work provides a generalized framework for leveraging pre-trained models in determining the intricate landscape of tRNA modifications.

## Graphical Abstract

**Graphical Abstract:**
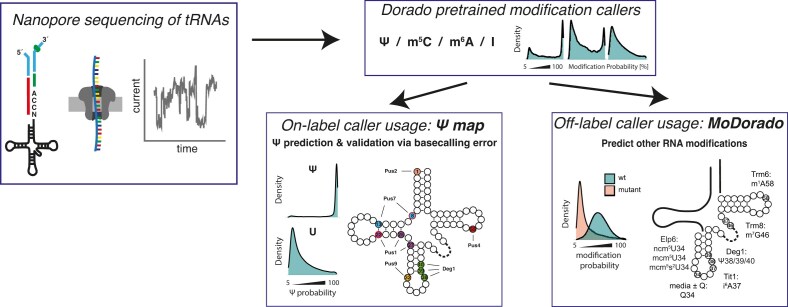

## Introduction

Transfer RNAs (tRNAs) play an elementary role in protein translation in all living cells. The correct function of tRNAs strongly depends on the chemical modification of individual nucleotides in the tRNA, which regulate tRNA folding, structure, stability, aminoacylation, and decoding [1]. Indeed, ∼100 different modifications are currently known in tRNAs, and each individual tRNA carries 5–15 modified sites [2, 3]. Modification levels at particular sites can vary, and in some instances, are dynamically regulated depending on external conditions [4]. Additionally, certain tRNA modifications are interdependent, an aspect that remains understudied [5, 6]. Importantly, defects in tRNA modifications cause human diseases (termed “RNA modopathies”) such as mitochondrial diseases, neurological disorders, and cancer [7]. Therefore, having simple and cost-effective methods to measure tRNA modifications at single-molecule resolution is crucial for elucidating disease mechanisms and may have diagnostic potential.

In recent years, high-throughput methods for transcriptome-wide detection of RNA modifications have advanced significantly, utilizing techniques such as chemical derivatization or antibody-based approaches combined with high-throughput sequencing. However, many of these methods present challenges related to selectivity and efficiency [8]. Hence, there is a need for a single, robust method to detect multiple modifications at the same time. A powerful approach for this purpose is to employ direct RNA sequencing (dRNA-seq) using nanopore sequencing from Oxford Nanopore Technologies (ONT), which has demonstrated accurate prediction of several types of modifications on a single messenger RNA transcript [9–11]. Although dRNA-seq was originally developed for long read sequencing, the method has been adapted to sequence short tRNAs [9, 12–16]. These adaptations involve ligating adaptors to the 5′ and 3′ ends of deacylated tRNAs, which are then threaded through membrane-embedded biological pores, where the nucleobases passing through a pore generate characteristic changes in the ionic currents across the pore. The distinct chemical properties of each nucleobase create unique signatures in the ionic current, allowing the inference of the sequence of the canonical A/C/G/U bases (termed “basecalling”).

So far, the methods for nanopore-based modification detection can be grouped into two main classes: the *sample comparison* methods and the *de novo* prediction methods. The sample comparison approaches detect modified bases by comparing modified versus unmodified RNA based on (i) characteristic deviations in the ionic current relative to canonical bases [17], or (ii) systematic basecalling errors that arise from these deviations [18]. On the other hand, the *de novo* prediction methods directly predict the modified RNA, which is achieved by the development of modification-specific models pre-trained on datasets in which the modification occurs within diverse sequence contexts [10, 19]. However, generating such comprehensive training datasets is technically demanding and remains infeasible for many RNA modifications.

In December 2023, ONT released a novel RNA-specific pore chemistry (SQK-RNA004, designated “RNA004” below), which showed accuracy improvement over previous iterations with the latest basecaller Dorado [20]. Subsequently, ONT released pre-trained modification calling models for the *de novo* detection of pseudouridine (Ψ) and N^6^-methyl-adenosine (m^6^A) in May 2024, and for 5-methyl-cytosine (m^5^C) and inosine in September 2024. These models were trained using synthetic RNA molecules containing modifications at specific positions within randomized sequence contexts (a strategy termed “randomers”). These randomer datasets in theory represent the highest possible quality training data achievable for modification detection models. However, the performance of these models beyond standard usage on synthetic RNA oligos, for instance on heavily modified tRNAs *ex cellulo*, remains to be determined [21].

**Figure 1.**
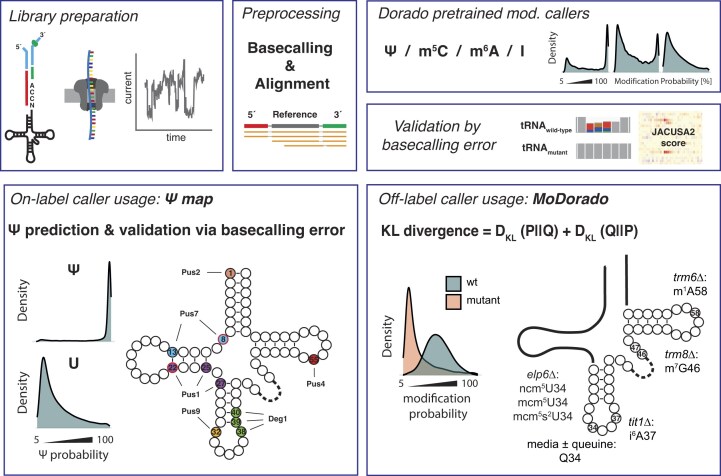
Strategy to determine tRNA modifications using nanopore dRNA-seq and MoDorado. For library preparation, splint adaptors were ligated to the 5′ and 3′ end of the deacylated tRNAs (top left). Raw data were basecalled with Dorado, and reads were aligned to the references using Parasail (top middle). The four pre-trained modification models for Ψ/m^6^A/inosine/m^5^C were used to predict modification probability for every nucleotide (plots show hypothetical examples of the distribution of probabilities). As validation of a modification, basecalling error differences between tRNAs from wt and strains lacking modification enzymes (mutant) were determined (top right). Using *pusΔ* strains, a near complete Ψ modification map was generated (“on-label usage”) and the responsible Ψ synthases assigned to individual Ψ sites (bottom left). To detect modifications beyond those included for model training (“off-label usage”), we developed MoDorado, an algorithm that compares prediction score distributions of pre-trained machine learning models using the KL divergence. By comparing wt and gene deletion strains, MoDorado detected seven additional modifications: ncm^5^U, mcm^5^U, mcm^5^s^2^U, m^7^G, queuosine, m^1^A, and i^6^A (bottom right).

As a naturally occurring class of RNA, tRNAs represent a good test case for the limits of RNA modification detection, since the sites of modification and the modification machinery in many cases are well known. Here, we present the first application of pre-trained modification-specific machine learning models to modification identification in a tRNA context (for overview, see Fig. 1). In a first step, we evaluated the Ψ caller for its ability to directly map Ψ on tRNAs from the fission yeast *Schizosaccharomyces pombe* (“on-label” usage, Fig. 1, lower left). The predicted Ψ positions were subsequently assigned to the pseudouridine synthases (Pus) using basecalling error differences between wild-type (wt) strains and strains with deletions in the seven respective genes, which revealed two previously unknown Ψ sites in *S. pombe* (Ψ8 and Ψ22) and indicated the existence of modification co-regulation circuits. Together, the combination of direct Ψ prediction and basecalling error analysis allowed us to generate a near-complete eukaryotic Ψ map. Moreover, we developed MoDorado (a modification detection algorithm based on pre-trained modification calling models in Dorado), which leverages prediction score distributions of existing machine learning models, a feature that so far has not been exploited in existing detection methods. MoDorado enabled us to detect seven additional modifications not used in model training, a strategy we coined “off-label use” (Fig. 1, lower right). Altogether, our study provides a powerful approach for the enhanced detection of tRNA modifications.

## Materials and methods

### *Schizosaccharomyces pombe* strain construction and growth

*Schizosaccharomyces pombe* strains used in this study are listed in Supplementary Table S1. Strains were grown in YES medium (5 g/l yeast extract, 30 g/l glucose, 250 mg/l adenine, 250 mg/l histidine, 250 mg/l leucine, 250 mg/l uracil, and 250 mg/l lysine) at 30°C. Synthetic queuine was supplemented to a final concentration of 0.1 μM as indicated. Strains from the Bioneer deletion collection were verified by amplification and sequencing of the bar code. Gene deletions of *deg1* (SPAC25B8.05), *pus4* (SPBC11C11.10), *pus7* (SPBC1A4.09), *tit1* (SPAC343.15), and *elp6* (SPBC3H7.10) were constructed using standard polymerase chain reaction (PCR)-based integration of an antibiotic resistance cassette replacing the gene of interest. Deletions were confirmed by PCR. Primers used for the generation of gene deletions are listed in Supplementary Table S2.

### Extraction of small RNAs

Small RNAs were isolated as previously described [13]. In short, *S. pombe* cells were cultured to an optical density at 600 nanometer (OD600) of 1, and 4 OD of cells were harvested by centrifugation. Cells were resuspended in 1 ml Trizol (Ambion) and 0.2 ml chloroform and glass beads were added. Cell lysis was performed by vortexing, followed by centrifugation at 16 000 rcf, 4°C for 15 min. After adding 215 μl ethanol to the upper phase, the sample was transferred to a spin cartridge [PureLink™ microRNA Isolation Kit (Invitrogen)] and centrifuged at 12 000 rcf for 1 min. Seven hundred microlitres of ethanol was added to the flow-through, and the sample was transferred to a new spin cartridge followed by centrifugation at 12 000 rcf for 1 min. After washing the cartridge with wash buffer, small RNAs were eluted with 50 μl of diethyl pyrocarbonate (DEPC)-treated water. Small RNAs were deacylated for 30 min at 37°C in 100 mM Tris–HCl (pH 9) prior to library preparation.

### Detection of inosine by Sanger sequencing

For the detection of inosine, small RNAs where extracted from wt *S. pombe* cells as described above. tRNAs with annotated I34 (Ala-AGC, Arg-ACG, Ile-AAT, Leu-AAG, Ser-AGA, Thr-AGT, and Val-AAC) were reverse transcribed with SuperScript IV (Thermo Fisher) using tRNA-specific reverse transcription (RT) primers (Supplementary Table S2), following the manufacturer’s protocol. Thereafter, the reverse transcription reaction was used as a template for cDNA amplification. Purified PCR fragments were subjected to Sanger sequencing, and inosine was detected by its characteristic A to G mutation (or T to C on the reverse strand).

### Generation of a library of representative *in vitro*-transcribed tRNAs from *S. pombe*

To obtain a mixture of representative, *in vitro*-transcribed tRNAs from *S. pombe*, a library of plasmids carrying the sequences of 46 *S. pombe* tRNAs was constructed. For this purpose, overlapping oligonucleotides (see Supplementary Tables S2 and 3) including a T7 promoter and a CCA tail were annealed, overhangs were filled in using Klenow polymerase and cloned into pJet1.2 using *Xho*I and *Nco*I (except *Sal*I/*Nco*I for Gln-tRNAs). Plasmids were amplified in *Escherichia coli*, and equimolar amounts of the plasmids were pooled to generate the plasmid library.

To generate *in-vitro* transcribed (IVT) tRNAs, the plasmid library was digested using *Xho*I and *Nsi*I and purified by agarose gel extraction, which resulted in the release of a pool of 115 nt T7-tRNA-CAA IVT templates. One microgram of template was used for IVT, which was performed at 37°C for 8 h using the TranscriptAid T7 High Yield Transcription Kit (Thermo Fisher Scientific). Samples were subsequently treated with DNaseI, and IVT tRNAs purified using phenol/chloroform/isoamylalcohol extraction followed by gel filtration on Sephadex G50 (GE Healthcare). tRNAs were treated with RppH (NEB) to generate 5′ monophosphorylated tRNAs, and samples were purified using the RNA clean & concentrate kit (Zymo Research).

### Library preparation for Nanopore Sequencing

In general, the preparation of tRNA libraries was carried out using the SQK-RNA002 or SQK-RNA004 kit (ONT; Supplementary Table S4) following the method previously described [12, 13] with minor modifications. Briefly, 100 pmol of deacylated small RNAs were refolded by heating, followed by ligation of the splint adapters using RNA ligase 2 (1× RNA ligase 2 buffer, 10% PEG8000, 2.5 mM ATP, 6.25 mM dithiothreitol (DTT), 6.25 mM MgCl_2_, 0.5 U/μl T4 RNA ligase 2, 20 pmol of each of the four splint adapters, 0.5 μl RNAse OUT) in a DNA loBind tube for 90 min at room temperature. Ligation reaction samples were subsequently separated on a 7 M urea/TBE PAGE gel (Tris-boronate-ethylenediaminetetraacetic acid, 8%), and ligation products were excised and purified using the ‘crush and soak’ method. The concentration of gel-purified ligation products was measured with the Qubit RNA HS Assay Kit (Invitrogen). Five hundred nanograms of ligated tRNAs were used for library preparation according to the standard dRNA-seq ONT protocol for the respective kit (RNA002 and RNA004), and VATHS RNA Clean beads (Vazyme) were used for purification.

### Nanopore sequencing

Samples were sequenced on a MinION Mk1C device using MinION FLO-MIN106D R9.4.1 or FLO-MIN004RA flow cells (ONT). After priming the flow cell, 75 μl of prepared sequencing library was loaded onto the flow cell. Sequencing runs were controlled by the MinKNOW software (ONT, version 22.10.5). Sequencing was typically carried out for 12 h depending on the number of collected reads and active pores. For FLO-MIN106D R9.4.1, runs of the raw bulk data file were stored, and the raw nanopore current intensity signals were re-processed with adjusted sequencing settings, as previously described [16]. An overview of each dataset, the sequencing chemistry (RNA002 or RNA004) and other specifics are shown in Supplementary Table S4.

### Data processing

#### Reference curation

As reference sequences for *S. pombe* tRNAs, all unique mature tRNAs from GtRNAdb [22] and all unique mitochondrial tRNAs from Ensembl Fungi [23] were combined (total: 86 sequences), and the 5′ and 3′ splint adaptor sequences were added. Since mitochondrial tRNAs showed low sequence coverage, they were excluded from further analysis.

#### Annotation of pseudouridine and other tRNA modifications in S. pombe

Ψ annotations and other tRNA modifications are derived from the scientific literature and are either a direct demonstration of modification at a given site in *S. pombe*, or Ψ is inferred from *Saccharomyces cerevisiae*. The individual sites and sources of information are given in Supplementary Fig. S1 and Supplementary Table S5.

#### Basecalling and modification calling with Dorado

Base calling was performed using the official basecaller Dorado version rna002_70bps_hac@v3 (RNA002) and version rna004_130bps_sup@v5.1.0 (RNA004). For RNA004 data, the option to perform modification calling with the four currently available RNA modification models (Ψ/m^6^A/I/m^5^C) was used. For each individual read, the modification callers assign a discretized prediction score with integer value from 0 to 255 (representing a predicted probability of modification from 0 to 1; 0%–100%) at positions of the corresponding canonical base (e.g. the Ψ model will only output scores for basecalled U positions). Only scores passing a threshold (default – 5%, which is discretized to a prediction score of 255 × 0.05 = 12) are written to output.

#### Read alignment and filtering

For alignment to the reference, all reads were taken and aligned to the reference using the optimal local sequence aligner Parasail (version 2.6.0) [24], as described [13]. The output of Parasail are all-against-all read to reference alignments, which were filtered in the following steps: (i) for each read, only the reference(s) with the highest Alignment Score (“AS”) were included; (ii) reads with AS <50 were excluded (threshold chosen according to the strategy described earlier [13]); and (iii) only full length reads were included.

### *De novo* detection of Ψ using the Dorado Ψ model

**Figure 2.**
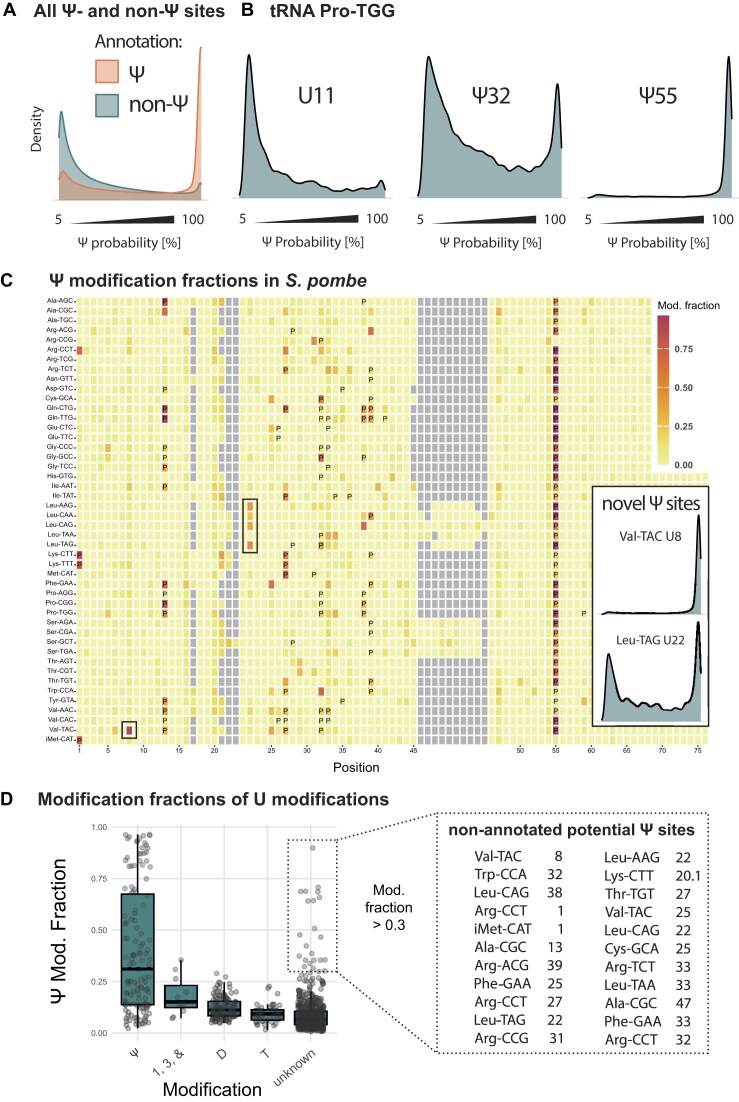
Determination of pseudouridine modification sites in tRNA from wt *S. pombe* reveals novel Ψ sites at positions 22 and 8. (**A**) Distributions of Ψ prediction scores of all annotated Ψ sites compared to all sites that are not annotated as Ψ of all tRNAs (“non-Ψ”). Annotated Ψ sites generally have higher Ψ probability scores than Us that are not annotated as Ψ; 5% is the default minimum threshold in Dorado. For visualization, the *y*-axis (“Density”) shows the frequencies of the discrete Ψ prediction scores smoothed using kernel density estimation. (**B**) Example distributions for annotated Ψ sites (32 and 55) and a non-Ψ site (U11) in tRNA Pro-TGG. (**C**) Plot of modification fractions (N reads with scores > 204/N all reads) on individual *S. pombe* tRNAs. The *x*-axis denotes tRNA position; tRNAs are annotated on the *y*-axis. The colour indicates the modification fraction. Annotated Ψ sites are marked with P. Boxes around sites refer to inlets: Distribution of probability scores from Ψ basecalling for the so far unannotated positions U8 (top) and U22 (bottom). (**D**) Bulky modifications at U34 and dihydrouridine (**D**) cause erroneously high Ψ fractions, and so far unannotated positions reveal likely Ψ modification. 1, mcm^5^U; 3, mcm^5^s^2^U; &, ncm^5^U. Nonannotated sites with high modification fraction (>0.3) are given on the right as potential novel Ψ sites. The position 20.1 on Lys-CTT refers to an inserted position based on multiple sequence alignment.

As basecalled reads contain errors, the positions with modification prediction scores may not actually exist (i.e. insertions), or certain reference positions may not exist in the reads (i.e. deletions). Therefore, only probability scores of correctly aligned (i.e. matched) positions were extracted (mismatched positions do not have a prediction score for the reference canonical base). Based on the prediction score distributions of Ψ-modified and nonmodified U positions (Fig. 2A), a prediction score threshold of 204 (which is equal to 255 × 80%) was set. This threshold was used to define the fraction of reads with Ψ modification (N _reads with scores> 204_/N _all reads_) for each U position.

To make the binary decision on a U site to be Ψ-modified or nonmodified with the Dorado Ψ model, the threshold for the Ψ fraction was chosen by plotting the percentage of annotated Ψ sites versus non-Ψ sites which can be determined for a certain Ψ fraction value (Supplementary Fig. S2). As the result, a Ψ fraction of 0.2 was used as a cut-off for Ψ detection *de novo*.

### Modification detection from basecalling errors with JACUSA2

As an orthogonal method, JACUSA2 (version 2.0.4) [18] was used for modification detection based on basecalling errors on aligned reads. JACUSA2 outputs a single score per position, and modifications at positions with scores above the outlier detection threshold from the nonparametric Tukey’s fences method were determined:


(1) \begin{eqnarray*}
{{x}_{outlier}} > \ {{Q}_3} + k*IQR
\end{eqnarray*}

where Q_3_ is the third quartile, IQR is the interquartile range and k = 1.5. The resulting scores were further filtered (≥10) to facilitate data evaluation. The cut-off is based on the distribution of non-U base calling error outliers.

### Modification detection with MoDorado

The main objective of MoDorado is to leverage the output of existing pre-trained modification callers (e.g. Dorado) to detect modifications, by comparing the prediction score distributions between samples. The prediction scores of Dorado take discrete integer values from 12 to 255, which can be highly uneven in samples with lower coverage (a minimum coverage threshold of 100 per tRNA was used). Therefore, the score distributions were first smoothed by constructing frequency histograms with a fixed number of bins (n_bins = 10) of equal spacing between 11 and 256. To compare the similarity of the smoothed prediction score distributions between samples, the Kullback–Leibler (KL) divergence for discrete probability distributions was used, which is given by equation 2.


(2) \begin{eqnarray*}
{{D}_{{\rm KL}}}\ (P\ ||\ Q) = \ \mathop \sum \limits_{x\ \in\ X} P\left( x \right){\rm log}\frac{{P\left( x \right)}}{{Q\left( x \right)}}
\end{eqnarray*}

where P and Q represent the smoothed histogram distributions of the wt and the mutant samples, respectively. As the KL divergence are nonsymmetric, the order of input distributions $P$ and $Q$ affect ${{D}_{{\rm KL}}}$. In MoDorado, the sum of ${{D}_{{\rm KL}}}\ (P\ ||\ Q)$ and ${{D}_{{\rm KL}}}\ (Q\ ||\ P)$ as a symmetric version of the KL divergence was used. Specifically,


\begin{eqnarray*}
\ {\rm Symmetric\ KL\ divergence}\ = \ {{D}_{{\rm KL}}}\ (P\ ||\ Q) + {{D}_{{\rm KL}}}\ (Q\ ||\ P)
\end{eqnarray*}

### Signal visualization

During basecalling, the option –emit-moves was turned on to have Dorado output a signal segmentation (commonly known as the “move table”). The move table segmentation assigns each base of a read to a segment of signals stored in the pod5 files. Since basecalled reads contain errors, such as insertions and deletions, only the signals of aligned reference positions (matches and mismatches) were extracted for visualization. As signals are variable in length (i.e. dwell time) and the signal lengths per base are always multiples of 6 (the fixed “stride” parameter in the RNA004 basecalling model), a subsample of six data points with equal spacing were taken for each base position. Lastly, to offset the intrinsic signal differences between two samples, we computed for each tRNA, (i) the differences in median signal intensity for each position between samples, and then (ii) the median of these differences across all positions on the tRNA, which resulted in a single scaler number and was used as a vertical shift parameter to add to the signals of the mutant sample.

## Results

### Direct Ψ calling in tRNAs reveals novel Ψ sites at positions 8 and 22

To investigate the utility of the Dorado Ψ caller on eukaryotic tRNAs, we isolated tRNAs from wt *S. pombe* and sequenced them using dRNA-seq (RNA004). Raw sequencing data was basecalled with Dorado using the Ψ model, followed by read alignment with Parasail [24] and filtering. The Dorado Ψ model assigns a prediction score to individual U bases within each read ranging from 0 (low probability) to 255 (high probability) and only outputs scores above the default threshold of 12 (corresponding to 5% of 255) (see the ‘Materials and methods’ section). Plotting the Ψ probability scores for all sites annotated as Ψ-modified (Supplementary Table S5 and Supplementary Fig. S1) showed a high peak near 100% probability, which was not observed for the U sites without Ψ annotations (Fig. 2A). Two independent biological replicates showed high reproducibility (R^2^ = 0.984; Supplementary Fig. S2A). As an example of individual sites in a tRNA, in Pro-TGG, Ψ55 [25] showed a unimodal distribution with a high peak near 100% Ψ probability, while the nonmodified U11 had mostly low Ψ probability scores, which agrees with the lack of Ψ annotation at U11 (Fig. 2B). Interestingly, Ψ32 had a bimodal distribution pattern, but also many intermediate values. A bimodal distribution may indicate partial modification [25], or may suggest that the prediction of Ψ is less accurate in certain sequence contexts. As will be shown below, intermediate prediction scores at true Ψ sites can arise from neighbouring modifications that affect the performance of the Ψ caller. Ψ probability scores at all U55 and U13 positions with Ψ annotation showed a bimodal distribution in some tRNAs, which might indicate partial Ψ modification in those tRNAs (Supplementary Fig. S2C).

The distinct distribution patterns of the Dorado Ψ caller at different sites highlight the need for a robust strategy to distinguish between Ψ-modified, nonmodified, partially Ψ-modified, or otherwise modified sites. To summarize a single prediction distribution, we defined a “Ψ modification fraction” for each given position as (N reads with scores > 204/N all reads) (see the ‘Materials and methods’ section) and plotted the fractions for each tRNA (Fig. 2C; each line represents a single tRNA, individual tRNAs are annotated on the *y*-axis). In general, the sites with high Ψ fractions were in good agreement with the annotated Ψ sites (Fig. 2C and D). Notably, there were also some previously nonannotated positions with a high Ψ fraction. One example is position 8 in Val-TAC (Fig. 2C, upper inlet), which has been described to be Ψ-modified in human embryonic stem cells [26], but not in yeast. Furthermore, position 22 in Leu-AAG, -CAA, -CAG, and -TAG contained a high Ψ fraction (Fig. 2C, lower inlet; tRNA-Leu-TAA has A22 and therefore no Ψ22). To our knowledge, this is the first description of Ψ modification of position 22 in tRNA (see below for verification).

Other nonannotated tRNA sites with high Ψ fractions were observed at positions that are known to be Ψ-modified in other tRNAs (1, 13, 25, 31, 32, 38, 39, Fig. 2D). Notably, several U modifications other than Ψ resulted in elevated Ψ fractions with the Dorado Ψ basecaller, in particular dihydrouridine (D) and the complex U34 modifications 5-carbamoyl-methyluridine (ncm^5^U), 5-methoxycarbonylmethyluridine (mcm^5^U) and 5-methoxycarbonylmethyl-2-thiouridine (mcm^5^s^2^U) (Fig. 2D), indicating that the Ψ model erroneously calls these modifications as Ψ. Altogether, the Dorado Ψ caller showed good correspondence with annotated Ψ sites and identified two new Ψ sites in *S. pombe* at positions 8 and 22.

### Assignment of predicted Ψ sites to known pseudouridine synthases reveals Pus1-dependent Ψ22 and Pus7-dependent Ψ8

The above analysis showed that U modifications other than Ψ can produce signal changes that yield increased probability scores by the Ψ caller (Fig. 2D) [27]. Therefore, to validate the predicted Ψ sites, we aimed to identify the pseudouridine synthases responsible for their modification. In *S. pombe*, there are seven known tRNA Pus enzymes: the cytoplasmic tRNA Pus enzymes Pus1 [28, 29], Deg1 [30], Pus4 [31], Pus7 [32], and Pus9 [25], as well as Pus2 and Pus3 (see Supplementary Fig. S3 for a phylogenetic tree) [33]. In *S. cerevisiae*, the Pus2 homolog localizes to mitochondria [34], whereas in *S. pombe*, high-throughput studies have reported Pus2 to be nuclear and Pus3 to be cytoplasmic [35]. However, these observations have not been followed up with detailed characterization, and the precise subcellular localization and functions of these proteins remain poorly understood.

**Figure 3.**
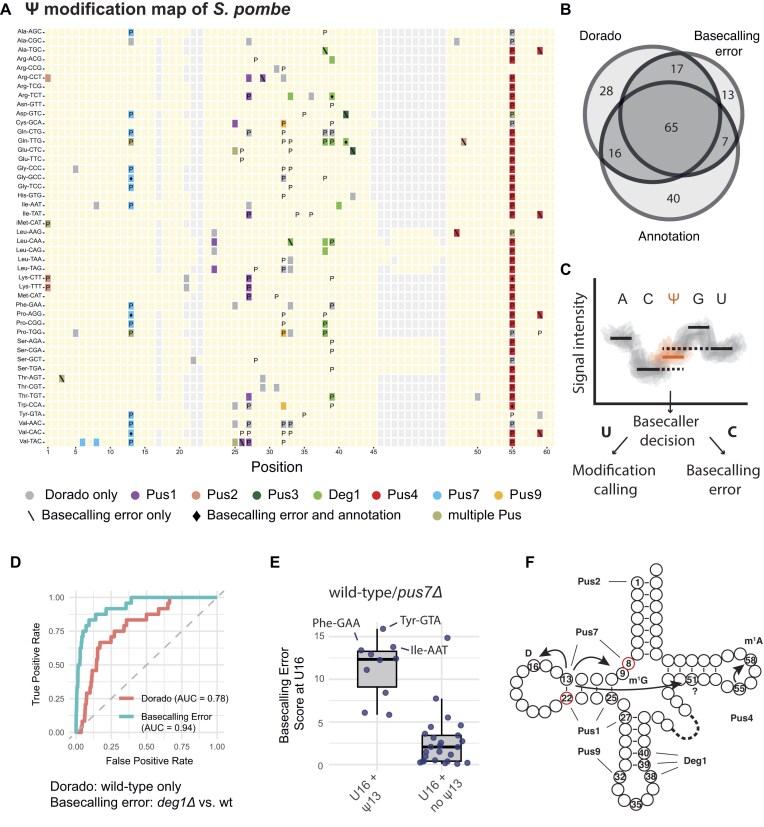
Determination of Ψ sites for pseudouridine synthases and their tRNA modification circuits in *S. pombe*. (**A**) Pus modification map in *S. pombe*. tRNAs are indicated on the *y*-axis; the *x*-axis denotes tRNA position. Annotated Ψ sites are marked with P. Ψ sites designated by direct Ψ basecalling and assigned to a pseudouridine synthases based on basecallling errors are shown in colour. Sites that are only found by direct Ψ basecalling, but not assigned to an enzyme by basecalling errors, are shown in grey. Sites that are designated Ψ based on basecalling errors alone are marked with a backslash (\). Sites that are assigned by basecalling and annotated but not identified using direct Ψ basecalling are marked with a diamond (♦). (**B**) Venn diagram showing the overlap between the Ψ sites from three categories: Dorado Ψ calling, basecalling error and annotation. (**C**) Schematic of the theoretical signal space of a hypothetical 5-mer and the Dorado decision process. The basecaller Dorado first decides whether a base should be called as U, and only then, the Ψ caller will be applied to produce a prediction score. Thus, basecalling errors will not yield a corresponding Ψ prediction. (**D**) Receiver operating characteristic (ROC) curves for direct Ψ calling at Deg1-dependent Ψ positions 38, 39, and 40, with Dorado (from wt) and basecalling error (from wt/*deg1Δ)*. (**E**) Potential cross-talk between Pus7-dependent Ψ13 and D16. Elevated basecalling errors were observed at D16 in tRNAs with, but not without, Ψ13 (*P*= .0015, unpaired two-sided *t*-test). (**F**) Overview of Ψ modifications on cytoplasmic tRNA in *S. pombe* and assignment of pseudouridine synthases from this study. The novel sites Ψ8 and Ψ22 are marked in red. Supernumerary circles depict positions 20.1 and 20.2. The potential modification crosstalk between Ψ13, D16, m^1^G9, and position 51 are shown with arrows. The known modification circuit between Ψ55 and m^1^A58 that was also detected here is indicated with an arrow. For modification crosstalk, see also the supplementary discussion and Supplementary Fig. S13.

To identify which Pus enzymes modify which sites, we compared basecalling errors from dRNA-seq of tRNAs between wt and strains carrying deletions in the respective Pus genes using JACUSA2 (Supplementary Fig. S4–10 and Supplementary Table S4) [18]. Ψ sites were assigned by defining an outlier threshold for the basecalling error scores (see the ‘Materials and methods’ section; Supplementary Fig. S11). To classify a site as Ψ-modified or unmodified using the Dorado Ψ model (i.e. to convert the continuous prediction score into a binary call), we determined the optimal Ψ fraction threshold by plotting the proportion of annotated Ψ versus non-Ψ sites correctly identified at varying threshold values (Supplementary Fig. S2B). This showed a 63% prediction percentage of annotated Ψ sites and 8% of non-Ψ sites predicted at a Ψ fraction of 0.2, which was subsequently used as a cut-off for Ψ detection *de novo* (i.e, with only the wt sample). Using this Ψ fraction cut-off of 0.2 resulted in 126 sites being called *de novo* by the Dorado Ψ model. Of these, 81 (64%) were previously annotated as Ψ. Furthermore, 82 sites (65%) could be assigned to a Pus enzyme based on the basecalling error score (Fig. 3A and B). A total of 20 sites were assigned solely based on basecalling errors, but not by direct Ψ calling with Dorado. Of these sites, seven had previously been annotated as Ψ. Importantly, the Ψ model and the error-based detection method draw from separate and complementary data sources: the Ψ model only outputs predictions for positions that were correctly basecalled as U, whereas the error approach relies on reads in which U was miscalled as another base (Fig. 3C). Lastly, 40 annotated sites, many located in the anticodon loop, were not detected by either Dorado or error profiling. This is likely due to the presence of many complex modifications in the close vicinity of Ψ sites (see below), or may be the result of mis-annotation.

**Figure 4.**
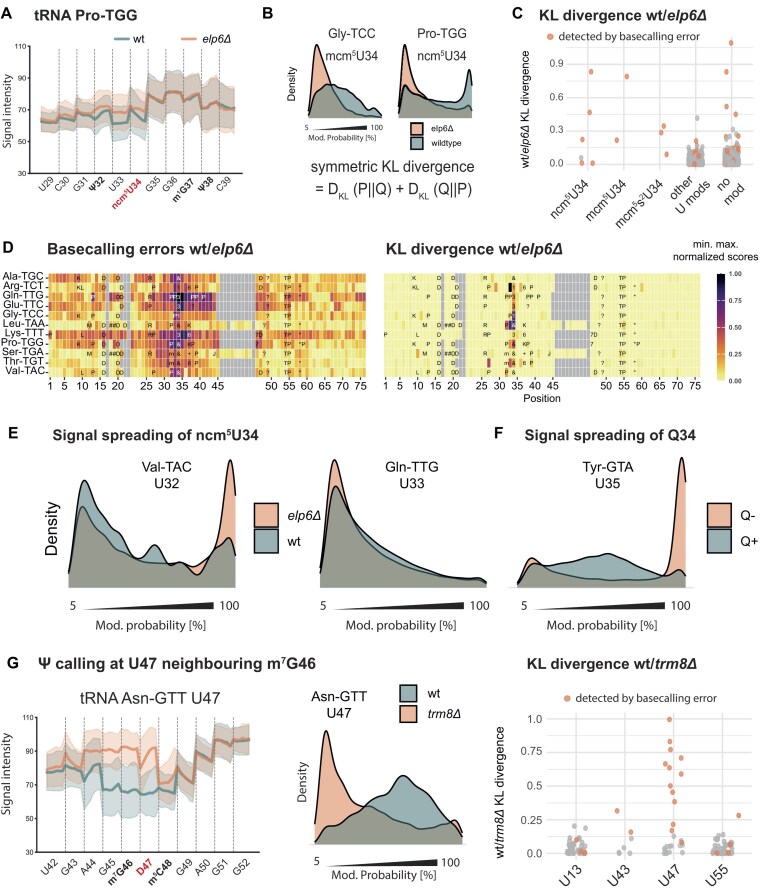
Off-label use of the Dorado Ψ caller to detect other U modifications (“MoDorado”), and detection of m^7^G by the Ψ caller at a neighbouring U. (**A**) Raw signals for U29-C39 for tRNA-Pro-TGG from wt and *elp6Δ*, which carries ncm^5^U at position 34. Solid lines represent the mean signal intensity at a given position across reads. The shaded area represents one standard deviation of the signal intensity. (**B**) Modification probability distributions, left: abnormal distribution for U34 in tRNA Gly-TCC, right: distribution for U34 in tRNA Pro-TGG. Representation as in Fig. 2A. Bottom: definition of the symmetric KL divergence. (**C**) KL divergence scores comparing the *elp6Δ* and wt strains at positions with Elp6-dependent tRNA modifications (ncm^5^U, mcm^5^U and mcm^5^s^2^U), other U modifications, and nonannotated sites. Sites also detected by basecalling errors are shown in colour. (**D**) Heat Map visualizations of basecalling error scores (left) and KL divergence scores (right) for tRNAs with Elp6-dependent U34 modifications. Scores were min–max normalized for comparison. (**E**) Prediction for Ψ32 in tRNA-Val-TAC in the presence (wt) or absence (*elp6Δ*) of U34 modification (ncm^5^U). (**F**) Prediction of Ψ35 in tRNA-Tyr-GTA in the presence (+Q) or absence (−Q) of Q34 modification. (**G**) Detection of m^7^G47 based on Ψ probability at the neighbouring U47. Left: Raw signals of tRNA Asn-GTT U42–G52 from wt and *trm8Δ*. Middle: m^7^G46 leads to an abnormal distribution of Ψ prediction at U47. Right: KL divergence scores at U positions. U47 is next to m^7^G46. U positions with increased basecalling errors in wt/*trm8Δ* are shown in colour.

An integrated view of the assignment of Ψ sites to Pus enzymes leads to the following conclusions (Fig. 3A and Supplementary Fig. S12): Pus7 modifies positions 6, 8, and 13 in *S. pombe*. For position 13 in the D arm, this is consistent with prior knowledge of Pus7 [32]. Dependence of Ψ8 on Pus7 is consistent with the fact that U8 is in UN**U**AR, the consensus motif of Pus7 [36]. Pus1 modifies positions 25 and 27 in the D arm of tRNAs [28, 29]. The newly identified Ψ22 site in the D arm is modified by Pus1. Pus9 modifies position 32 in the anticodon loop [25]. Deg1, which is the direct homolog of Deg1/Pus3 from *S. cerevisiae* and humans, modifies positions 38, 39, and 40 in the anticodon stem [30]. Pus4 modifies U55 [31]. Pus2 modifies position 1 in three tRNAs, and Pus3 modifies iMet-CAT at position 1. The limited number of identified targets for Pus2 and Pus3 is unexpectedly low, raising the possibility that Pus2 and Pus3 may act on mitochondrial tRNAs or other classes of cellular RNAs that have not yet been characterized.

To compare the performance of the Dorado Ψ model (*de novo* prediction from wt *S. pombe*) and the basecalling error approach (comparing wt strains with strains deleted for *pus* genes), we computed the area under the ROC using *deg1Δ*, which lacks Ψ38, Ψ39, and Ψ40. The error approach had a higher area under the curve (AUC) (0.94) than Dorado (0.78) (Fig. 3D), showing that *de novo* Ψ prediction remains challenging in tRNAs. In addition, the error approach can detect cross-modification crosstalks. As a point in case, we observed increased basecalling errors in wt compared to *pus4Δ* not only at the expected Ψ55 [31], but also at position 58 (Supplementary Fig. S8 *)*, which is N^1^-methyl-adenosine (m^1^A)-modified by Trm6/Trm61 [37], thus confirming the known Ψ55-m^1^A58 modification circuit [38]. Similarly, we observed basecalling error patterns indicative of Pus7-dependent Ψ13 inhibiting Trm10-mediated m^1^G9 [39] and enhancing Dus1-dependent D16 modification [40] (Fig. 3E and Supplementary Fig. S13, see also supplementary discussion). Figure 3F provides a summary of all Pus sites and their associated crosstalk described in this study.

### Off-label use of pre-trained modification callers to detect other tRNA modifications

Our above analysis indicated that U modifications other than Ψ can produce distinctive signal signatures in nanopore sequencing that cannot be cleanly assigned by the Dorado Ψ caller to either U or Ψ (Fig. 2B and C). We reasoned that these distinctive modification signals may also create distinctive patterns in the model’s prediction scores, and thus, that the Ψ prediction scores might contain features that allow detection of these other modifications, even though the model was not specifically trained for their identification. This motivated us to develop MoDorado, a tool to detect diverse types of modifications beyond those supported by modification-specific Dorado models, a strategy that we coined “off-label use”. As a “sample comparison” method, MoDorado compares the distribution of prediction scores at a particular position between a modified RNA (i.e. wt) and an unmodified control (such as RNA from a gene deletion strain, or IVT RNA). To assess the (dis)similarity between probability score distributions of wt and mutant, we defined the symmetric KL divergence as a distance metric for modification detection between samples, with high KL divergence indicating the presence of modification (see the ‘Materials and methods’ section). To obtain samples lacking a specific modification, dRNA-Seq was performed on four gene deletion strains (*elp6Δ, tit1Δ, trm8Δ*, and *trm6Δ*), as well as on a wt strain grown in medium without queuine, which therefore lacks queuosine 34 (Q34) modification [41] (Fig. 1, lower right). All sequencing datasets are summarized in Supplementary Table S4.

To test the off-label prediction strategy, we first compared the tRNAs from a wt strain and an *elp6Δ* mutant, which lacks the Elongator complex required for performing several U34 modifications (ncm^5^U, mcm^5^U, and mcm^5^s^2^U) [42, 43]. Raw signal intensities as well as Dorado Ψ probability scores showed clearly distinguishable patterns between wt and *elp6Δ* at U34 (Fig. 4A and B; see Supplementary Fig. S15 for basecalling errors wt/*elp6Δ*), and distribution of Dorado Ψ scores differed from that of validated Ψ sites by exhibiting a greater proportion of intermediate values (c.f. Fig. 4B to Ψ55, Fig. 2B). Applying the KL divergence method to wt compared to *elp6Δ*, elevated KL divergence values were observed at the expected U34 positions that carry Elp6-dependent tRNA modifications (Fig. 4C), demonstrating that the Dorado Ψ caller is sensitive to uridine modifications other than only Ψ. The KL divergence values differed between different tRNAs, which may be due to different sequence contexts of the U34 modifications, different modification levels, or may be due to other modifications in the vicinity (Supplementary Fig. S14A). Comparing KL divergence to basecalling error scores showed that these U34 modifications led to increased error scores with a strong spreading effect of false positive values both upstream and downstream of U34 (Fig. 4D, left), while the KL divergence score was centred around U34 and its direct neighbouring bases (Fig. 4D, right).

Importantly, the elevated KL divergence at the close neighbours of modified U34 indicates that modification signatures can influence the predictions at neighbouring nucleotides. To investigate this, we compared the Ψ probability scores at U32 of tRNA-Val-TAC, which is annotated as Ψ32 [25], but lies close to the Elongator-modified ncm^5^U34. Indeed, Ψ32 was not detected in wt *S. pombe* by the Dorado Ψ caller, nor was it detectable by basecalling errors (Fig. 3A). In *elp6Δ*, however, the Ψ prediction scores showed a clear Ψ signature at position U32, suggesting that U32 is Ψ-modified, and that ncm^5^U34 impairs direct Ψ calling at U32 (Fig. 4E, left panel). It is also possible that U32 is only Ψ-modified in the absence of ncm^5^U34, a possibility that cannot be distinguished at this point.

Similarly, a Ψ35 that lies close to Q34 of tRNA-Tyr-GTA, was only identified in the absence of the queuosine modification (Fig. 4F), and Q34 caused erroneous Ψ calling at some U32 sites (Supplementary Fig. S14B). These results are consistent with the reduced ability of the Ψ model to detect Ψ *de novo* in the anticodon loop (Fig. 3A), because signal interference by other (bulky) modifications such as Q or ncm^5^U in the vicinity spreads across multiple nucleotide positions, which currently presents a limitation of modification detection using nanopore sequencing. Of note, some other annotated Ψ33 sites (e.g. Gln-TTG, Fig. 4E, right panel) showed no difference in prediction scores in *elp6Δ*, suggesting possible mis-annotations.

Currently, there is no Dorado model available for G modifications. Given the observation that current signals from one modification can spread into a neighbouring U position, we investigated whether off-label use of the Ψ model could be employed to detect G modifications that lie next to a U. We therefore sought to detect Trm8-dependent m^7^G46 [44] by computing the KL divergence of Dorado Ψ probabilities at the neighbouring U47 (which is annotated as D47 [40]) in wt and *trm8Δ* (see Supplementary Fig. S16 for basecalling errors wt/*trm8Δ*). Indeed, m^7^G caused signal distortion at and around position 46, which resulted in an elevated KL divergence score at U47 (Fig. 4G), showing that U-neighbouring G modifications can be detected with “off-label use” of the Ψ model.

### MoDorado integrates multiple modification callers into an expanded tRNA modification map

**Figure 5.**
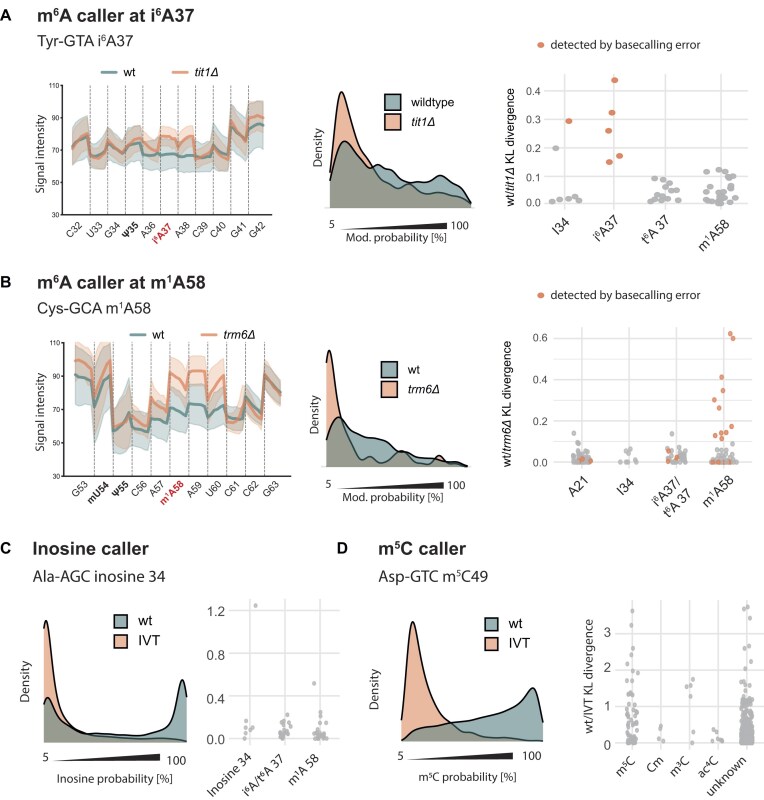
Evaluation of the m^6^A, inosine and m^5^C models on tRNAs. (**A**) Off-label detection of i^6^A37 with the m^6^A model in wt/*tit1Δ*. Left, raw signals of tRNA-Tyr-GTA C32 to G42 in wt and *tit1Δ*. Representation as in Fig. 4A. Middle, modification probability distributions generated by the m^6^A caller at A37 of tRNA-Tyr-GTA. Representation as in Fig. 2A. Right, KL divergence at A37 and other A positions in wt/*tit1Δ*. Positions with elevated basecalling errors are shown in colour. (**B**) Off-label detection of m^1^A58 with the m^6^A model in wt/*trm6Δ*. Left, raw signals; middle, modification probability distributions; right, KL divergence scores. Representation as in panel (A). (**C**) Evaluation of the inosine model. Left, inosine probability distributions from wt and IVT tRNA-Ala-AGC inosine 34. Right, KL divergence scores at known inosines and other A positions. (**D**) Evaluation of the m^5^C model. Left, m^5^C probability distribution at the known m^5^C49 position in tRNA-Asp-GTC. Right, KL divergence scores at known m^5^C sites and C position carrying other modifications.

Since the Dorado Ψ model exhibited broad sensitivity towards other U and neighbouring G modifications, we next evaluated the remaining three Dorado modification models (m^6^A, inosine and m^5^C) for their utility in detecting other tRNA modifications. Conveniently, since m^6^A itself is absent from tRNAs [1], all adenosine modifications detected by the m^6^A model by default constitute “off-label use” of the model. We investigated Tit1-dependent N^6^-isopentenylated A37 (i^6^A) [45] and Trm6/Trm61-dependent m^1^A58 [37, 46] by analysing dRNA-seq of tRNA from *tit1Δ* and *trm6Δ* strains (see Supplementary Fig. S17 for basecalling errors). The m^6^A model resulted in elevated KL divergence at i^6^A37 positions in wt/*tit1Δ* (Fig. 5A) and at m^1^A58 in wt/*trm6Δ* (Fig. 5B), thus establishing off-label use of the m^6^A model for the detection of both i^6^A and m^1^A.

Inosine and m^5^C are known to exist in several *S. pombe* tRNAs [47–50], thus allowing the evaluation of the Dorado inosine and m^5^C models. Since the adenosine deaminase Tad2/Tad3 required for inosine formation is essential for viability in *S. pombe* [48], we created a library of IVT tRNAs as unmodified tRNAs in order to be able to determine KL divergence scores, since this approach requires the comparison of modified and unmodified samples. Furthermore, annotated inosine modifications were verified by reverse transcription and Sanger sequencing, which confirmed their presence in all seven previously annotated tRNAs (Supplementary Fig. S18A). Interestingly, an increased KL divergence score for inosine was only found at one of the seven known inosine sites (Fig. 5C), showing that the performance of the Dorado inosine caller is suboptimal in the context of tRNAs. Other nearby modifications likely interfered with the detection, because inosine 34 in Ser-AGA was correctly detected in the absence of i^6^A at position 37 (*tit1Δ*; Supplementary Fig. S18B).

The levels of m^5^C in tRNAs from *S. pombe* are well known from earlier work [49]. The KL divergence scores were elevated at many of these sites (Fig. 5D), but m^5^C detection with the m^5^C model was not as reliable as Ψ with the Ψ model. Furthermore, 3-methyl-cytosine (m^3^C) also showed elevated KL divergence values using the m^5^C model, but since this modification is found in the anticodon loop and we did not cross-verify the scores with the respective gene deletion or another orthogonal method, it is possible that the m^5^C model senses another adjacent modification, rather than m^3^C itself.

**Figure 6.**
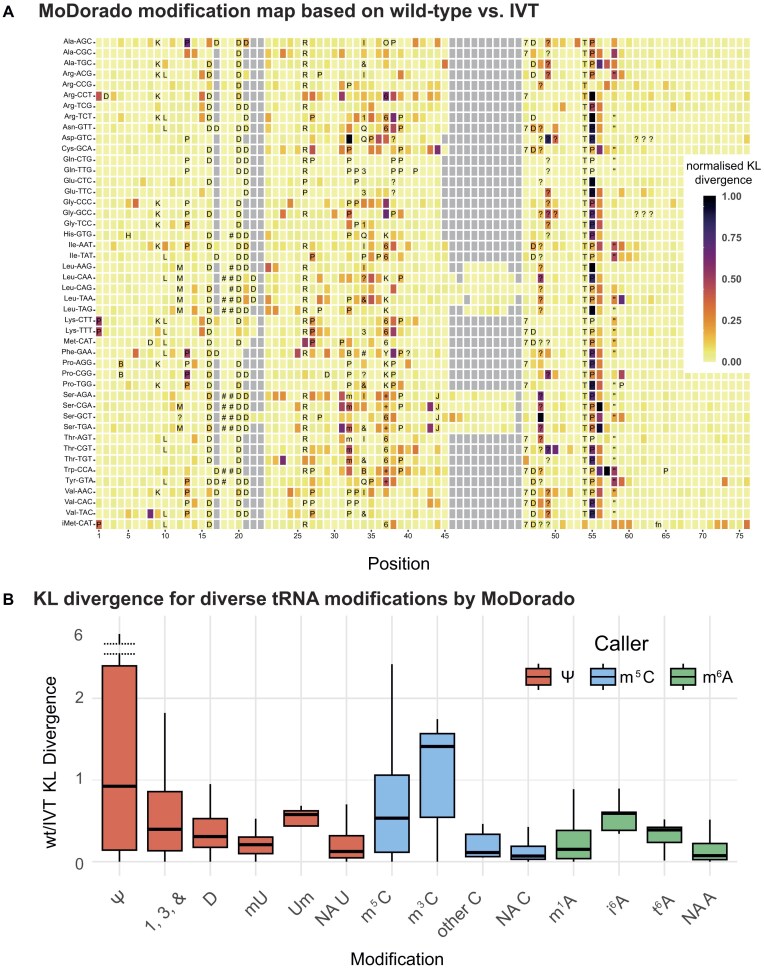
(**A**) MoDorado integrates multiple models for an expanded tRNA modification map. Visualization of KL divergence scores between wt and IVT tRNAs in *S. pombe* using the Ψ, m^5^C, and m^6^A models for U, C, and A positions, respectively. Known and predicted modification sites (Supplementary Table S5) are marked using the unified RNAMods code for modified residues as in the Modomics database [3]. (**B**) KL divergence of various modifications in *S. pombe* tRNAs (wt/IVT). Colours represent the nucleotide and the respective modification model used.

Having evaluated the four Dorado modification callers, we next combined them into a single MoDorado framework to assign KL divergence scores to every A, U, and C base in the tRNAs (no G model exists yet), and we used this approach to produce an expanded dRNA-seq-based tRNA modification map. To this end, tRNAs from wt strains were compared to IVT tRNAs using the m^6^A/Ψ/m^5^C models for A/U/C sites, respectively (the inosine model was not used due to its limited performance). Figure 6A shows the normalized KL divergence for each tRNA at single-nucleotide resolution. While regions near the anticodon loop remain complex, higher KL divergence was generally observed at modified sites compared to nonmodified sites, especially for on-label modifications such as Ψ55 and m^5^C48/49. Multiple sites, especially A sites (e.g. position A23 in Ser-AGA and Thr-TGT or A15 in Arg-CCT and Lys-CTT), also showed high KL divergence but, to the best of our knowledge, no modifications have previously been observed at those positions. An overview of KL divergence scores comparing wt and IVT RNA shows that the m^6^A/Ψ/m^5^C models sense a variety of modifications (Fig. 6B). In summary, MoDorado, which combines the on- and off-label usages of pre-trained modification callers, offers a promising approach towards generating an expanded tRNA modification map.

## Discussion

The intricate network of modifications on tRNAs, often in close proximity, makes their detection and characterization a technically demanding task. Existing approaches for their detection using nanopore-based technologies generally use two types of features, namely basecalling errors [51], signal changes [17], or a combination of the two [52]. In contrast, the MoDorado approach presented here uses as input the prediction scores of pre-trained modification-specific machine learning models, a feature that has not yet been exploited, but is readily available as part of basecalling with Dorado. This has allowed us to predict not only Ψ/inosine/m^5^C in tRNAs, but also other types of U/A/C modifications through the “off-label use” of these modification-specific callers, as well as G modifications by querying neighbouring bases. Altogether, MoDorado detected ncm^5^U, mcm^5^U, mcm^5^s^2^U, m^7^G, Q, m^1^A, i^6^A, and (potentially) m^3^C modifications. However, confident assignment of specific modifications with MoDorado requires prior knowledge, either from existing annotations or through comparative analysis showing loss of signal in modification-deficient samples.

In recent years, pre-trained modification-specific models have shown promising results of *de novo* detection for the now retired RNA002 sequencing chemistry, with the main focus on m^6^A [10, 19]. For the vast majority of RNA modifications, it remains infeasible (in some cases, impossible) to generate a training dataset with diverse sequence contexts for model training. On the other hand, unsupervised sample comparison methods are modification-unspecific, with the limitation that unmodified control samples (in form of gene deletions or IVT RNA) are necessary [17, 53]. Exploiting the fact that different modifications may show similar perturbations in the nanopore ionic current, the MoDorado work is an effort to bridge the two families of methods by repurposing and broadening the utility of *de novo* methods. As more pre-trained *de novo* models become available for the latest RNA004 chemistry and beyond and their performance continues to improve, further effort is warranted to understand their strengths and limitations in the diverse realm of (t)RNA modifications.

Among the modification callers available in Dorado, we found the Ψ caller to be the most consistent with previous Ψ annotations in tRNA, whereas the inosine and m^5^C callers showed lower consistency. Due to the high density and diversity of modifications, direct *de novo* modification calling remains challenging on tRNAs. On a similar note, using IVT tRNAs as unmodified controls in MoDorado produced less accurate modification maps (Fig 6A), as compared to gene deletion strains as controls. Since IVT tRNAs are completely devoid of any modifications, gene deletions strains, with majority of other modifications still intact, likely constitute a more appropriate control, since their tRNAs are more similar to wt than IVT tRNAs. However, bulky modifications at one site can hinder modification calling at nearby sites, thus compromising the performance of the modification caller. These limitations and challenges highlight the need for an integrative approach for both *de novo* and sample comparison methods, with the latter requiring a careful choice of unmodified controls.

Using a combination of methods, we have generated a tRNA pseudouridine modification map and have identified the respective pseudouridine synthases, thus assigning all enzymes to their Ψ sites (Supplementary Fig. S19 gives an overview of all newly assigned modifications in this study). This has led us to identify a novel target for Pus1, Ψ22 in four leucine tRNAs (tRNA-Leu-AAG, -CAA, -CAG, and -TAG). This site lies opposite position 13 in the D arm of tRNAs, a position that is frequently Ψ-modified [33]. However, the Ψ22-modified tRNAs have a G at position 13, suggesting that Ψ22 modification serves to enhance Wobble basepairing to G13 and thus to stabilize the tRNAs [54]. We also identified a Pus7-dependent Ψ modification at position 8 in tRNA-Val-TAC, a site that so far has not been described in *S. pombe* [26]. Furthermore, we have identified novel Ψ sites in specific tRNAs at positions that are annotated as Ψ-modified in other tRNAs, thus showing conserved positional modification across different tRNA species (Supplementary Fig. S19).

In summary, the off-label use of pre-trained modification callers by MoDorado provides a novel and powerful framework for the interpretation of direct (t)RNA sequencing data using nanopore sequencing. With the release of alternative pre-trained models and their performance improvements in the near future, we anticipate that the off-label prediction strategy will contribute to the comprehensive mapping of (t)RNA modifications, the identification of regulatory modification circuits, and the exploration of their impact on human health and disease.

## Supplementary Material

gkaf795_Supplemental_Files

## Data Availability

Sequencing data has been deposited in the European Nucleotide Archive (Accession number is PRJEB88080). The analysis scripts for the paper and the source code for the MoDorado tool are available at https://github.com/KleistLab/MoDorado. A frozen version is available at https://zenodo.org/records/15533642.
